# Dr. Kinglake, in Reply to Mr. Mantal, on Gout

**Published:** 1805-09-01

**Authors:** Robert Kinglake

**Affiliations:** Taunton


### *To the Editors of the Medical and Physical Journal.*

Gentlemen,

In my Observations on Mr. Mace's case is remarked, "that my sentiments on the nature and cure of gout did not essentially differ from those of your ingenious correspondent Mr. Mantal;*" that opinion was solely founded on his comments on Mr. Edlin's "account" of Mr. Baker's case. The previous communication of Mr. Mantal, published in No. 70 of your Journal, had at that time escaped my perusal, nor were his farther thoughts on the subject, inserted in No. 75, at all anticipated by me. It is necessary to premise these facts, to justify the irreconcilable difference which subsists between Mr. Mantal's notions of the nature and cure of gout, and those of my own.

Mr. Mantal has favoured the public with three distinct communications on the subject of gout in the course of five months, and which he wishes to have considered as supplemental to, and explanatory of, each other. These communications have gained by delay the practical value of

* Medical and Physical Journal, No. 74, p. 360.

of being in part illustrated by the occurrence of what the author deems demonstrative cases of the validity of the opinion which he espouses. Liberal discussion is the torch of truth, and cannot fail to elucidate the obscure speculations of physiological conjecture. Mr. Mantal has been largely indulged in expatiating on my theory and practice relating to gout, you will consequently feel no unwillingness in acceding to my right of ample reply. The subject is indeed important, but it shall be discussed by me with becoming brevity.

Mr. Mantal imagines insuperable objections to admitting that the genuine seat of gouty inflammation is in the ligamentous and tendinous structure, and affirms the theory that insists on this opinion to be a "*gratuitous assumption.*" But how does he prove it to be gratuitous? Not by exhibiting a view of the phenomena of the disease incompatible with such theory, but by roundly asserting it is gratuitous.

Permit me to ask Mr. Mantal, if every feeling and every external appearance in gouty inflammation do not concur to prove that the affection originates and chiefly continues on the ligaments and tendons? These parts are the principal instruments of loco-motion, they naturally occupy an intermediate office between muscle and bone; and to fit them for their purpose, they possess less excitability than the former, and more than the latter. They are susceptible of inflammatory affection, slow indeed in its approach, but becoming at length exquisitely severe. The structure of these parts is too dense easily to admit of suppurative inflammation, hence the period of pain and tension is in general indefinitely prolonged. The tardy accession of inflammatory disease on the ligamentous and tendinous fabric often insidiously disturbs the general excitability of the system, and occasionally induces rigor, followed by increased heat, indigestion, loss of appetite, bilious tinge of the secreted fluids, head-ach, and the usual oppressive sensations of symptomatic fever. As the local pain augments, the circulating fluids become more rapidly and equally distributed over the frame, and at length are more particularly determined to the cutaneous surface, where, by the consequent event of perspiration, much superabundant heat is dissipated and temporary relief obtained. This is a general view of the accession, progress, and termination of a gouty paroxysm. The short period of diminished excitement, which succeeds the incipient and more active stage of a gouty fit, has delusively induced duced the popular belief that the attack has been salutary, and that the affection is Nature's mode of expelling from the system either morbid matter or motion!

Mr. Mantal may remain incredulous as to the disease originating in the ligamentous and tendinous structure, but his want of credence does not alter the real state and character of the distemper, which every unprejudiced reasoner, and every gouty patient, will admit to be derived from the fabric insisted on. The analogy of common sprain to gouty inflammation is another argument which raises my claim to correctness, higher than that of a mere "*gratuitous assumption.*" When a ligament or tendon is mechanically forced beyond its healthful dimensions, what happens? Neither immediate inflammation nor inability to move the affected part. These effects usually occur in the course of a few hours after the accident, and mark the slow but eventually severe accession of inflammatory disease. Rigor also, and more or less of systematic ailment, often arise between the time of inflicting the external violence and that of the full production of its inflammatory consequence. During this period the latent growth of stimulant disease at the sprained part imperceptibly generates sympathetic commotion throughout the system, which, in the instance of gout, is erroneously supposed to have occasioned the local inflammation, because this appearance does not conspicuously attain its formal character before the systematic effects of its earlier stage are somewhat subsided. Thus a strong resemblance may be intelligibly made out between the inflammatory affection of the ligaments and tendons from mechanical violence, and that which spontaneously arises from an unnatural susceptibility in the motive power of these parts for being morbidly impressed, whether induced by the debilitating influence of immoderate loco-motive exertion, or proceeding from a distempered state of systematic excitability, preponderating on the ligamentous and tendinous structure.

"*Assumptions,*" however "*gratuitous,*" are not proved to be so by mere affirmation; that which is destitute of support should appear in its untenable deficiency, and not be rejected by declamatory refusal. When a theory is impugned, the public have an anxious interest at stake; it is at once hoped that the evils of an erroneous opinion will be prevented, and the advantages of superior correctness realised. But how has Mr. Mantal discharged this important obligation? He has objected to the ligaments and tendons tendons being the seat of gouty inflammation, to suggest his opinion that it is rather a "disease of the membranes that cover the joints." What are these membranes? My knowledge of anatomy reminds me of no membranous covering on the joints excepting the cellular texture which forms the interstitial or connecting fabric of the surrounding parts, but no where distinctly envelopes the joints. The bones constituting the joints, are variously tied and bound together by round and capsular ligaments, and more or less overspread by tendinous insertion or adhesion.

But Mr. Mantal removes all obscurity respecting his opinion of the nature of the *membrane,* on which he would have gout to originate, by likening it to the "membranous parts of the stomach and liver;" and conceives that, by analogy of structure, the community of gouty affections subsisting between the visceral and articular parts is clearly explicable. Mr. Mantal then, with seeming triumphant complacency, remarks, "perhaps Dr. Kinglake would find it difficult to assign a reason, why, when gouty inflammation attacks the ligaments and tendons of the great toe, of the ankle, and knee, in succession, or at the same time, the intermediate parts of that structure should escape without injury; but there seems no room for that objection on the view of the disease which I have presented." No more difficulty occurs in assigning a reason why an inflamed joint from gout should excite correspondent disease in a distant joint, than why extreme pain in the head or stomach should be occasionally reciprocated between these several organs; than why sprain should ever induce locked-jaw; than why external blistering should relieve visceral ailment; in short, than why the most common instances of remote sympathy should obtain.

It will not be denied that similar structure is actuated by kindred motive powers, and that diseased conditions may be either simultaneously or successively excited in distinct portions of it, through the medium of the connecting fabric, without necessarily involving the transmitting or intermediate parts in disease; an inflammatory action in the dense fabric of a ligament or tendon, may be propelled through the comparatively lax texture of muscular and cellular substance, and produce no diseased impression until it may reach the analogous structure of other joints. In like manner, remote sympathies obtain between the sufferings of other similar parts, independently of contiguous influence.

It

It is not a little strange that Mr. Mantal should raise a difficulty against my theory, to make way for one of his own, which he has founded on an *ideal membrane* existing only in his own imagination. The joints have no surrounding membrane resembling either the internal mucous lining, or the external peritoneal covering of the stomach, liver, or any other viscus. Why then will Mr. Mantal invite a dispute concerning a non-entity? Mr. Mantal first creates a *membrane,* distributes its extensions, and determines its sympathies; he then, not so *originally,* but quite as obscurely, assumes *torpor* on the vital organs, however resulting, as the cause of *inflammatory* gout.

The laws of Nature or (if not synonymous) those of Darwin, are variously pressed into the service of generating inflammation. At one time Mr. Mantal is obliged to suppose that *strength,* by a law of organic life, is the offspring of *weakness;* at another, that the protecting ordinance of Nature has made the place of *vital languor,* the post of *motive energy.* Visceral or constitutional *torpor,* Mr. Mantal asserts, is the cause of inflammatory gout on the joints. How is it conceivable, that while parts actuated by the higher energies of vital power should be from torpor (which implies comparative inaction if it signifies any thing) incapable of inflammatory irritation on a visceral part, but that it should be able to excite that disease on a remote joint? No reason appears to common sense intelligence, why this seemingly incompatible effect should happen; but Mr. Mantal has supplied this want of explanation by considering the process as under the provident guidance of the "*vires medicatrices naturæ,*" which cannot err, and which Mr. Mantal affirms, will defend the vital parts to the *last extremity,* by generating an inflammatory action on the joints, the salutary effects of which, are shewn in the removal of the morbid *torpor* of the system.

Has this theory a feature of the captivating simplicity, and dexterous sufficiency of Nature? Is it not toilsomely incumbered by the crooked and incompetent contrivances of art? Does it not bespeak a TORPID and fallacious attempt at a satisfactory explanation?

But how does the idea of *torpor* comport with the usually existing state of the system of those who are most afflicted with gout? Does not an attack commonly ensue a series of intemperance in eating and drinking, or some other stimulant excess? Does it arise after a course of abstemiousness, the occurrence of mental distress, or the influence of any other debilitating power? Is it not manifoldly nifoldly proved, that the *torpor* (if Mr. Mantal will have it) of extreme sobriety and temperance very generally prevents the periodical recurrence of gout?

Mr. Mantal has not done Dr. Darwin full justice in employing *torpor* as a cause of inflammatory excitement, and not resorting to his ingenious device for rendering its operation at all explicable, namely the accumulated excitability during the prevalence of *torpor,* by which the usual portion of vital power not being expended, additional susceptibility is supposed to be induced for being impressed by stimulant agents. This explanation is indeed more specious than real; it imagines the vital process to be undiminished in a state of *torpor,* and that its energy is accumulating in circumstances of *inaction!* Neither the one nor the other can intelligibly happen. If a part be torpid, it must be deficient in the necessary energy to generate an excess of vital power, and when vital power abounds, it irresistibly dissipates itself in the irregular actions of violent disease. Torpor therefore is too *negative* a state to be compatible with the *positively* active quality of inflammation, and much too barren ever to generate an exuberant portion of vital power.

Mr. Mantal, in dismissing his theory of gout, observes, that, "with respect to the practice of Dr. Kinglake, my only difference with him is, that I think *caution* is necessary in the application of the remedy." My advice in directing the treatment is replete with *caution,* on my principle of the nature of the affection. It is held by me to be solely a disease of excessive temperature; and when either threatened by uneasiness, or characteristically shewn by violent inflammation and pain, that it ought to be resisted by the topical application of cold water, and that the practice should be unremittedly prosecuted as long as a painful degree of heat might continue to prevail on the affected part; that when it shall cease, the remedy should be suspended, and resorted to as often as the recurrence of inflammatory excitement might require, attending to any ailment which might either accidentally or sympathetically arise in the system, according to its seeming nature. If tremor and coldness should be felt in the region of the stomach, relief should be attempted by stimulant agents; if heat and pain, by small draughts of cold aqueous liquids at short intervals. Under this obvious caution, at once suggested by common sense, and capable of being precisely regulated by the sure indications of natura tural feeling, no fear need be entertained respecting the perfect safety and eventual efficacy of the treatment.

Mr. Mantal thinks the disease should never be prevented; but that "when the inflammation is completely formed on the extremities, and not at the first feeling of painful stiffness of the affected joint, and when visceral uneasiness arising from torpor (which he believes always precedes the inflammaton of the extremities) has entirely subsided, the cooling remedy might be safely resorted to." This may be a judicious caution, if the theory on which it is founded be just; but surely that is too factitious to be natural, too much composed of "*gratuitous assumptions*," of *torpor* and *action*, of native providence, and of hypothetical laws of organic life, to pretend to an indisputable claim to intelligent assent. It is farther opposed by the evidence of facts. The forming stage of the disease has, in numerous instances, been counteracted, and in spite of the powerful stimulus of *visceral torpor* aided by this law, or that, and farther backed by the "*vires medicatrices naturæ*," has not again advanced, and all his seeming repulsive mischief has manifestly rather relieved than disordered the general health.

The precarious connection subsisting on Mr. Mantal's theory of *inflammatory torpor* between the torpid viscus and the inflamed joint, and the importance which is made of relieving the former by promoting the latter, rather justifies than condemns the stimulant treatment of gout, by rendering the safety of the refrigerant practice in every instance extremely problematical. In the doubtful calculation, whether or not the inflamed joint (the offspring of *visceral torpor)* has endured long enough to have sufficiently energised the system, too much well founded anxiety must ever be felt to admit of a confident and successful use of the remedy. With such theoretical shackles, therefore, it is scarcely conceivable that any beneficial reform could be effected in the old mode of treating gouty inflammation.

Mr. Mantal's view of gout is embarrassed with much too large a portion of *contingent* mischief to obtain many proselytes to his *cautionary* treatment. When acted upon, it must necessarily lead to a timid, feeble, undecided, and often inert practice. The delicate balance of *visceral torpor* and *inflamed extremities* must ever tremble in the patient's fearful imagination, and resolutely lead him back to *patience* and *flannel*, rather than to purchase relief at such speculative risk.

Mr.

Mr. Mantal then refers to a case of sprain, treated by cold applications, inducing sympathetic gout in the stomach, and terminating in death. This is egregiously stretching an attempt alternately to destroy by sympathetic *torpor* and sympathetic *inflammation*. After affirming that *visceral torpor* always precedes gouty inflammation on the joints, and that the latter removes the former, it is hardly appropriate to his argument that a sprained, and of course inflamed ankle, had been too speedily relieved of its salutary pain by the influence of topical cold, and had given occasion to sympathetic gout, or a state of fatal torpor in the stomach. Here a very arbitrary association is made to arise; it did not result from habit. The chain of irritability said to subsist between the stomach and ankle, was not as usual first disturbed by *torpor* at the stomachic link, but inflamed at that of the ankle; and though Mr. Mantal asserts that this chain runs through the *same membranous structure*, yet, by a mysterious but preserving law of life, its *visceral end*, when suffering from gouty excitement, is in a state of torpor, whilst its *articular extremity* is tortured by inflammatory action! Such inconsistent arguments may be convenient to hypothetical reasoning, but will not satisfy the just demands of scientific inquiry.

Mr. Mantal's theory, in my opinion, does not bear him out in explaining the phenomena of the disease, as he appears to imagine; but its supposed validity is not refuted by my considering it as groundless. It must be tried by the evidence of facts, and farther experience will furnish a true award of its merits. It is utterly beyond the reach of my conception, and at variance with my uniform observation, that visceral excitement when at all connected with articular or gouty inflammation, should be founded in torpor. The viscera are highly susceptible of original as well as sympathetic inflammation, why therefore should gouty disease, said to be diffused throughout the system, especially attack the dense structure of the joints, and fail to produce inflammatory effects on the vital organs?

My theory, be it good or bad, (and it cannot be justly contended that my "*vanity*," as Mr. Mantal questions, has led me to suppose it perfect) accounts for every possible diversity of systematic disorder sympathetically arising from the severe local irritation which prevails, by attributing it to the existing circumstances of vital power in the organs which may become affected. The sympathetic effect of acute and protracted pain may be shewn in either an an inflammatory or a nervous form, and will be respectively cognizable by the re-active symptoms that prevail.

The ground of the different symptoms will be laid in the comparative temperature and energy of vital motion. Inflammatory tone and action will mark the one, whilst tremulous debility and chilliness will distinguish the other; the precise degree of either, will be governed by the temperamental conditions of motive power.

Mr. Mantal exhibits, in his second communication on Mr. Baker's case, a lucid and instructive view of the refrigerant treatment of gout. By the removal of visceral torpor, and the establishment of systematic tone through the efficiency of articular inflammation, he arrives at practical rules, nearly similar to those which are derived from the changeful course of temperature on the principles of my doctrine. In my mode of reasoning, it is vindicable to counteract the full formation of gout by topical cold; this Mr. Mantal disapproves, but he justifies the unremitted use of the remedy after it has fully obtained. We shall therefore practically agree in the vast majority of instances, nor will any disinclination or "*reaitancy*," (as Mr. Mantal terms it) be shewn by me in admitting the due import of whatever further experience may prove to be adverse to my present view of the subject.

Mr. Mantal, in his third communication, commenting on the cases adduced by Mr. O'Neal and Messrs. Scott and Taynton, discovers a precipitancy and warmth of expression unworthy of his usual correctness and moderation. His philanthropy has probably misled him to hasty and severe remonstrance. It were illiberal to suspect him of a worse motive. He conceives my theory of gout to be deeply involved in the cases detailed by Mr. O'Neal, and Messrs. Scott and Taynton, and thinks it should be revised and corrected, to prevent the practice founded on it, from falling into disrepute and consequent disuse. It would indeed be gratifying to me to clear the question of every difficulty in the way of solid truth. No undue tenaciousness of opinion on my part shall ever obstruct such a desirable elucidation; but Mr. Mantal will have the candour to exempt me from censure, in not allowing my acquiescence to precede my conviction.

The case of Mr. Baker has not in my judgment received any confirmation from the others on which Mr. Mantal comments, as affording corroborative evidence. Mr. O'Neal's case cannot, with either common consistency or ingenuousness, be dragged into the discussion of the merits of the cooling cooling treatment of gout, the practice pursued on that unhappy occasion was unprecedented, supported by no authority, and vindicable only by the author's peculiar knowledge of circumstances. It was instituted for an ideal disease as it respected the visceral affection, and perhaps no conditions of either health or distemper could escape the destructive influence of such deadly coldness.

Mr. Macer's case, as reported by Messrs. Scott and Taynton, does by no means warrant Mr. Mantal in inferring any thing to the disadvantage of my theory of gout in support of his own; yet he has with too much apparent captiousness availed himself of it, as disreputable to the indiscriminate use of topical cold in gouty inflammation. This case has already been remarked on by me in No. 74 of your Journal, but probably in a way not satisfactory to Mr. Mantal; but how can it be impartially considered as bearing at all unfavourably on my want of sufficient caution, when its unbiassed authors remark as follows? "We forbear making any comments on the above facts, but it appears worthy of remark, that this gentleman, in former fits of the gout, had suffered as much constitutional indisposition when the parts affected were wrapped up in flannel as under the present (cooling) mode of treatment." This avowal, it may be presumed, shifts a heavier *onus probandi* on Mr. Mantal, in establishing his assertion that the injury resulted from the unseasonable use of topical cold, than he appears to imagine; and the entire silence with which this important fact is passed over, presents a regretful instance of Mr. Mantal discovering more solicitude to confirm his own speculation than fairly to solve the question at issue. Nor does Mr. Mantal's *playful* recurrence to the several terms, "*accidental,*" "*casual,*" and "*co-incidencies,*" at all contribute to the philosophic value of his discussion.

It surely will not be gravely contended that these terms have no admissible significancy in philosophical inquiries, and that they are, as Mr. Mantal would insist on, "*declarative of ignorance.*" It is evident to the most superficial observer, that the regular progress of fever is often disturbed by the accidental occurrence of inflammatory irritation; that the variolous and vaccine diseases may be deranged in their course; in short, that no ailment is exempt from the disconcerting influence of *accident, casualty,* and *co-incidence.* But to be more pointed, may not the bursting of a blood-vessel in a vital organ, the occurrence of an habitual epilepsy, an occasional apoplexy, the first first impression of febrile contagion, or that of various other adventitious evils to which human life is incident, pending a paroxysm of gout, prove mortal without incurring any just imputation on the credit of a mode of cure adopted for the disease?

These occurrences are too rare to be liable to mislead; they are also the effects of certain causes, and might be totally irrelevant either to the definite action of a given disease, or to the operation of remedies instituted for its cure. Can Mr. Mantal imagine a fact in either the moral or physical world that is not liable to have its accustomed efficiency or character diversified by contingent influence? or will he aspire to a severity of theory that shall explain every anomaly, systematise every irregularity, and present one unvaried picture of uniform agency? If he cannot, why will he contend that Mr. O'Neal's case could not have suffered from the *co-incident* effects of an unauthorised treatment; and that Mr. Mace's does not present a *casual* instance of inveterate association, which neither protracted nor curtailed gout could destroy? Why does he attempt to raise on forced and loose analogies a theory which cannot endure but on a basis of more correct inquiry and more just conclusions? These are the errors of intemperate zeal, they are the crude offspring of visionary physiology, and are chiefly objectionable for the premature attempt they discover to controvert a different opinion on equivocal and insufficient data.

No harsh epithet Mr. Mantal has thought proper to bestow on me, shall be retorted on him; he has my implicit credit for integrity and honest intention, nor do his strictures on my theory at all displease me, they serve to invite farther inquiry, and will probably conduce additionally to elucidate a subject of momentous and arduous investigation. Experience has been hitherto my sole guide in this research, no other source of instruction can so safely and usefully advance my knowledge on the subject; and to no other authority, however specious, will my persuasion of the correctness of my opinion, respecting the refrigerant treatment of gout, ever yield.

Mr. Mantal, and all others who may laudably occupy their attention on the subject, must proceed on experimental ground, examine with scrupulous fidelity the facts which may occur, and neither raise any constructions, nor draw any inferences from them, which do not legitimately flow from their undisguised and true import. To inquiries thus chastened, the public may confidently look for sterling truth; and whether the result should be propitious or adverse to my opinion, it shall have my unprejudiced and cordial assent. I am, &c.

*Taunton, May* 20, 1805. ROBERT KINGLAKE.

